# Deletion of *Cdkn1b* in ACI rats leads to increased proliferation and pregnancy-associated changes in the mammary gland due to perturbed systemic endocrine environment

**DOI:** 10.1371/journal.pgen.1008002

**Published:** 2019-03-20

**Authors:** Lina Ding, Lauren B. Shunkwiler, Nicholas W. Harper, Yang Zhao, Kunihiko Hinohara, Sung Jin Huh, Muhammad B. Ekram, Jan Guz, Michael J. Kern, Alexander Awgulewitsch, James D. Shull, Bart M. G. Smits, Kornelia Polyak

**Affiliations:** 1 Department of Medical Oncology, Dana-Farber Cancer Institute, Boston, Massachusetts, United States of America; 2 Department of Medicine, Harvard Medical School, Boston, Massachusetts, United States of America; 3 Department of Pathology and Laboratory Medicine, Medical University of South Carolina, Charleston, South Carolina, United States of America; 4 Department of Regenerative Medicine and Cell Biology, Transgenic and Gene Function Core, Medical University of South Carolina, Charleston, South Carolina, United States of America; 5 Department of Medicine, Transgenic and Gene Function Core, Medical University of South Carolina, Charleston, South Carolina, United States of America; 6 Department of Oncology, School of Medicine and Public Health, University of Wisconsin-Madison, Madison, Wisconsin, United States of America; St. Jude's Children Research Hospital, UNITED STATES

## Abstract

Mammary epithelial progenitors are the normal cell-of-origin of breast cancer. We previously defined a population of p27^+^ quiescent hormone-responsive progenitor cells in the normal human breast whose frequency associates with breast cancer risk. Here, we describe that deletion of the *Cdkn1b* gene encoding the p27 cyclin-dependent kinase inhibitor in the estrogen-induced mammary tumor-susceptible ACI rat strain leads to a decrease in the relative frequencies of Cd49b^+^ mammary luminal epithelial progenitors and pregnancy-related differentiation. We show by comprehensive gene expression profiling of purified progenitor and differentiated mammary epithelial cell populations that p27 deletion has the most pronounced effects on luminal progenitors. *Cdkn1b*^*-/-*^ females have decreased fertility, but rats that are able to get pregnant had normal litter size and were able to nurse their pups implying that loss of p27 in ACI rats does not completely abrogate ovarian function and lactation. Reciprocal mammary gland transplantation experiments indicate that the p27-loss-induced changes in mammary epithelial cells are not only caused by alterations in their intrinsic properties, but are likely due to altered hormonal signaling triggered by the perturbed systemic endocrine environment observed in *Cdkn1b*^*-/-*^ females. We also observed a decrease in the frequency of mammary epithelial cells positive for progesterone receptor (Pr) and FoxA1, known direct transcriptional targets of the estrogen receptor (Erα), and an increase in phospho-Stat5 positive cells commonly induced by prolactin (Prl). Characterization of genome-wide Pr chromatin binding revealed distinct binding patterns in mammary epithelial cells of *Cdkn1b*^*+/+*^ and *Cdkn1b*^*-/-*^ females and enrichment in genes with known roles in Notch, ErbB, leptin, and Erα signaling and regulation of G1-S transition. Our data support a role for p27 in regulating the pool size of hormone-responsive luminal progenitors that could impact breast cancer risk.

## Author summary

The frequency and proliferation of tissue-specific stem and progenitor cells is associated with the risk of malignancy. Thus, regulators of the pool size and proliferation of progenitor cells determine cancer risk. p27 is a key regulator of cellular proliferation and is required for the terminal differentiation of a number of cell types. Here we show that genetic deletion of *Cdkn1b* in ACI rats susceptible to estrogen-induced mammary tumors decreases the relative fraction of Cd49b^+^ luminal progenitors identifying p27 as a key regulator of the proliferation and pool size of these cells. Progesterone, acting via the progesterone receptor (Pr), is an important regulator of mammary epithelial cell proliferation and differentiation. Based on ChIP-seq we found that Pr targets different sets of genes in *Cdkn1b*^*+/+*^ and *Cdkn1b*^*-/-*^ mammary epithelium and that this is associated with differences in proliferation and differentiation states. Thus, p27 regulates breast cancer risk and tumor development via regulating the pool size and hormonal-responsiveness of luminal progenitors.

## Introduction

Emerging data indicate that breast epithelial stem cells and progenitors are the normal cells-of-origin of breast carcinomas and factors that influence breast cancer risk (e.g., reproductive and hereditary factors) may alter the number and/or properties of these cells [1]. Besides germline mutations in cancer-predisposing genes including *BRCA1* and *BRCA2*, the most significant determinants of breast cancer risk are reproductive history and mammographic density. A single full-term pregnancy in early adulthood decreases the risk of estrogen receptor-positive (ER^+^) postmenopausal breast cancer, the most common form of the disease [2]. By characterizing normal human breast epithelial cells, we previously found that the most significant parity-associated gene expression and epigenetic changes are observed in CD44^+^ progenitor-enriched cells and the molecular profiles of these cells was also significantly different in *BRCA1* and *BRCA2* mutation carriers compared to healthy age and parity-matched controls [3]. Thus, alterations of this progenitor-enriched cell population may explain the cellular and molecular basis of the breast cancer-preventive effects of pregnancy. *CDKN1B*, encoding the p27 cyclin-dependent kinase inhibitor [4], was among the top differentially expressed genes showing lower levels in parous compared to nulliparous women as well as in controls relative to *BRCA1* carriers. The relative number of p27^+^ mammary epithelial cells was also lower in parous compared to nulliparous and control versus *BRCA1*-mutant women. Furthermore, the frequencies of p27^+^ and Ki67^+^ cells varied and inversely correlated during the menstrual cycle implying that a subset of these cells represents quiescent and proliferating hormone-responsive breast epithelial progenitors, respectively. Our analysis of mammary epithelial cells in mice of different ages and reproductive stage corroborated our findings in humans and also suggested that p27 may be required for the proliferative quiescence of hormone-responsive mammary epithelial progenitors [5].

The breast cancer-protective effect of pregnancy is also observed in rodent models of breast cancer and can be mimicked by hormonal manipulations such as high doses of estrogen, estrogen and progesterone, or human chorionic gonadotrophin (hCG) [6–8]. Most of these studies have been conducted in rats using chemical carcinogens (i.e., 7,12-dimethyl-benz[a]anthracene or N-methyl-N-nitrosourea) to induce mammary cancer [9]. Carcinogen-induced mammary tumors in rats are ovarian-hormone dependent (70% ER^+^; 30% ER^-^), but these carcinogens are not implicated in human breast cancer although several of the mutations initiated by them, such as those in *PIK3CA*, are also commonly observed in human breast tumors [10, 11]. A rodent model of breast cancer that closely resembles the genotype and phenotype of estrogen-dependent human breast tumors involves estrogen (i.e. 17β-estradiol; E2)-induced tumors in the ACI inbred rat strain [12–14]. An attractive feature of the ACI rat model is the generation and characterization of genetic backcrosses with the mammary tumor-resistant Brown Norway (BN) inbred strain, which recently allowed for the detailed mapping of mammary tumor genetic susceptibility loci [15, 16]. Several of these loci are orthologous to regions of the human genome that have been identified as breast cancer susceptibility loci based on GWAS (genome-wide association study) studies, highlighting the relevance of the E2-induced ACI rat mammary tumor model to human breast cancer [13]. However, such susceptibility loci were also identified using DMBA-induced mammary tumor models [17], suggesting that both carcinogen and estrogen-induced models reflect at least some aspects of the human disease, thus, both are useful models for preclinical studies.

Genetic deletion of *Cdkn1b* in mice leads to increase in body size and organs without obvious morphological abnormalities with the exception of dysfunctional ovaries resulting in female sterility [18]. Prior studies in mice investigating the role of p27 in hematopoietic and neural stem and progenitor cells have determined that p27 is a key regulator of quiescence in transit-amplifying progenitors but not in stem cells [19, 20]. However, the role of p27 in mammary gland development has been controversial. One study reported that p27 is required for mammary gland morphogenesis and function [21], while another described that loss of p27 has no discernable effects on mammary morphogenesis and differentiation [22]. A potential explanation for these seemingly contradictory findings could be due to the abnormal ovarian function and infertility of female *Cdkn1b*^-/-^ mice [18, 23] necessitating mammary fat pad transplantation assays to assess mammary gland development.

The mammary gland is a unique organ as most of its development occurs postnatally driven by ovarian and pituitary hormones [24]. Although mammary gland development has most extensively been studied in mice and certain species-specific differences exist, the essential roles for ovarian and pituitary hormones, including estrogen, progesterone, prolactin, and growth hormone are universal [25]. Estrogen (E2) and E2-responsive cells are driving proliferation and ductal elongation during puberty, while in adults progesterone is the more predominant mitogen. Pregnancy-associated lobulo-alveogenesis and subsequent lactation are driven by the combination of high levels of estrogen, progesterone, and prolactin. Some of the actions of these hormones is direct only affecting the cells with receptors for them, while others, especially the proliferative effects, are indirect, mediated by paracrine mechanisms. For example, amphiregulin and RANKL is essential for estrogen and progesterone-induced mammary epithelial cell proliferation, respectively [25].

Rat mammary tumor models are widely recognized as the superior rodent model in reflecting clinical features of human ER^+^ breast cancer, however, reverse genetics approaches have historically been limited due to the inability to routinely modify the rat genome. While targeted rat genome manipulation technologies have been available since the early 2000s [26], recent development of the CRISPR-Cas9 gene editing technology has revolutionized the generation of genetically engineered rat models for human traits [27]. We adopted the CRISPR-Cas9 technology to inactivate the *Cdkn1b* gene in the rat genome. We transferred the loss-of function mutations to the E2-induced mammary tumor-susceptible ACI rat strain, which allowed us to test the role of p27 in hormone-responsive mammary epithelial progenitors in this model with high relevance to the human disease.

## Results

### Generation and characterization of *Cdkn1b* knockout rats

**Fig 1:**
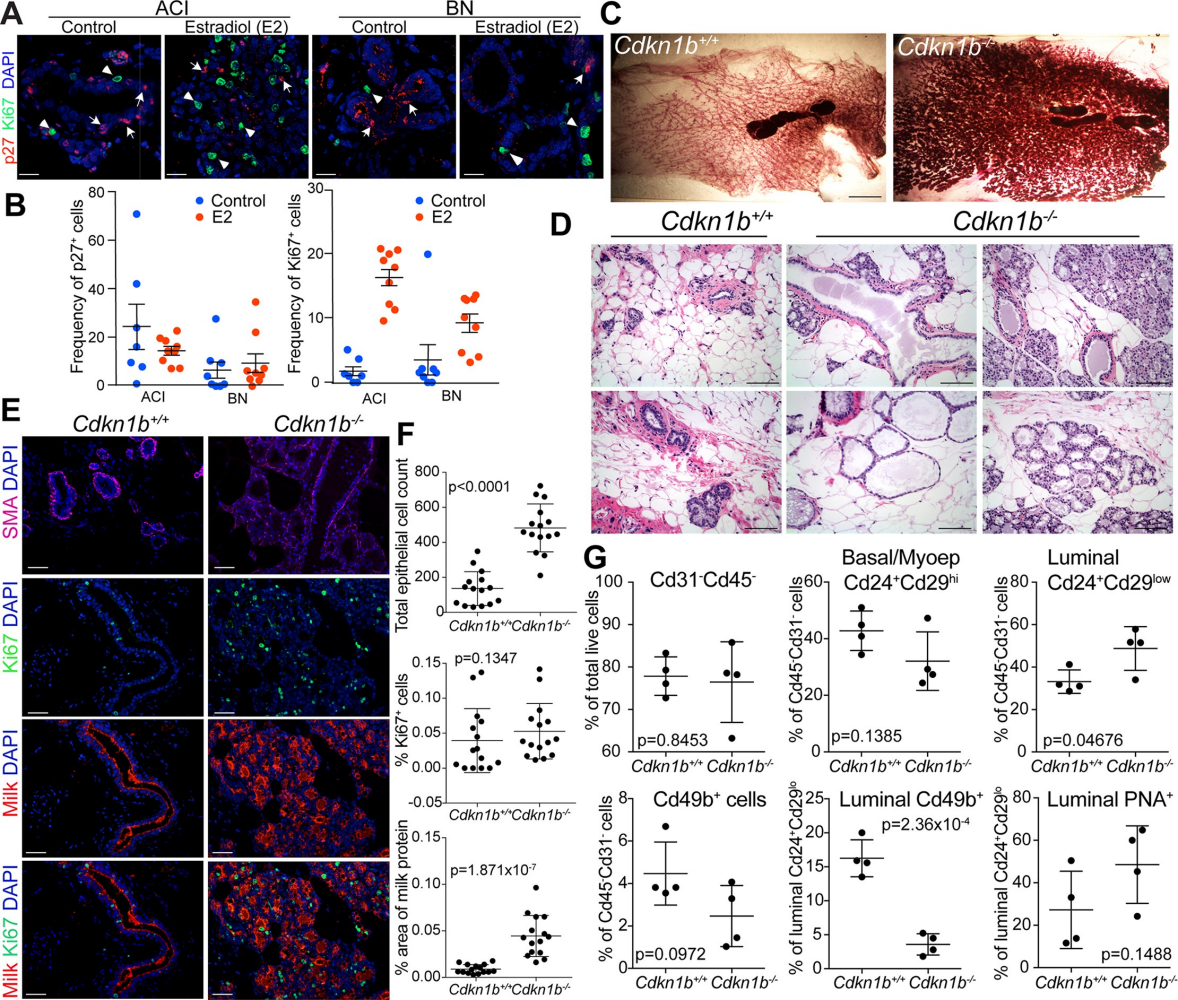
The role of p27 in rat mammary gland. (A) Multicolor immunofluorescence analysis of p27 and Ki67 expression and (B) quantification of p27^+^ and Ki67^+^ cells in mammary glands of control and estrogen-treated ACI and BN rats (n = 2/group, 4–5 images/slide). Scale bar 25 μM. Error bars represent mean ±SEM. The frequency of p27^+^ cells is significantly different between ACI and BN rats (p = 0.0087), and the frequency of Ki67^+^ cells is significantly different between control and E2-treated groups (p = 3.4x10^-7^) (arcsin transformation, two-way ANOVA with post hoc t test). (C) Whole mounts of inguinal/abdominal mammary glands of 9-week-old females. Scale bar 10 mm. (D) Hematoxylin-eosin staining of mammary glands of 9-week-old female *Cdkn1b*^*+/+*^ and *Cdkn1b*^*-/-*^ rats. Scale bar 150 μM. (E) Immunofluorescence analysis of the indicated markers in the mammary glands of 9-week-old female *Cdkn1b*^*+/+*^ and *Cdkn1b*^*-/-*^ rats. Scale bar 75 μM. (F) Quantification of the indicated cell types and fractions. Error bars represent mean ±SD. Statistical significance determined using Welch two sample t test of arcsin transformed values. (G) Frequency of the indicated cell populations in mammary glands of 9-week-old female *Cdkn1b*^*+/+*^ and *Cdkn1b*^*-/-*^ rats. Error bars represent mean ±SD. Statistical significance determined using Welch two sample t test of arcsin transformed values.

First, we assessed if the frequency of p27^+^ cells is also associated with mammary tumor risk in rats by immunofluorescence analysis of mammary glands of E2-induced mammary cancer-susceptible ACI and resistant BN rats, without and after three weeks of E2 treatment. The frequency of p27^+^ cells was significantly higher in ACI than in BN rats and it decreased with E2 treatment only in ACI rats. In untreated control animals the relative abundance of proliferative Ki67^+^ epithelial cells was about the same in the two strains and it was significantly higher following E2 treatment in both strains with ACI rats showing a more obvious increase compared to BN rats (**Fig 1A and 1B**). These data suggest that ACI rats susceptible to E2-induced mammary tumors might have a higher number of hormone-responsive luminal progenitors with proliferative potential compared to BN rats and that p27 may regulate the quiescence of these cells in this model making it suitable for studying the role of p27 in mammary epithelial progenitors. However, larger numbers of animals would need to be analyzed to conclusively prove strain-specific differences.

Next, we utilized the CRISPR-Cas9 system to generate knockout mutations in the rat *Cdkn1b* gene. The single guide RNA (sgRNA) was designed to target exon 2 (the first coding exon) of the rat *Cdkn1b* gene (**S1A** and **S1B Fig**). Zygotes were derived from the Sprague-Dawley (SD) outbred rat strain, since specific inbred rat strains including ACI are lowly responsive to superovulation procedures, which renders working with ACI zygotes too inefficient. A total of 67 and 19 zygotes were injected in 2 batches, either with a solution containing a plasmid (pX330) expressing the *Cdkn1b*-targeting sgRNA and *Cas9* gene, or with a solution containing purified *Cdkn1b*-targeting sgRNA and Cas9 mRNA. From the injected zygotes, 47 and 15 were transferred to pseudopregnant females. The first batch resulted in a litter of 12 pups of which 3 showed mutations in *Cdkn1b* exon2. The second injection batch yielded a litter of 8 pups of which 1 showed a mutation in *Cdkn1b* exon2. Two mutations with the highest potential impact on *Cdkn1b* function were transferred through the germline showing a deletion mutation by PCR analysis (**S1C Fig**). Sequencing of the mutations revealed a 32-bp deletion (DEL-32) and a 65-bp deletion (DEL-65), both disrupting the open reading frame of the *Cdkn1b* gene (**S1B Fig**). We bred the selected mutations to the ACI inbred strain for 6 backcross generations.

We generated a cohort of *Cdkn1b*^*+/+*^ wild-type and *Cdkn1b*^*-/-*^ knockout rats at early (N1xN1) and later (N6xN6) generations for the characterization of the mutant phenotype. Western blot analysis of p27 protein levels using tissues from homozygous DEL-32 animals at 9 weeks of age confirmed the success of p27 knockout in multiple tissues including mammary gland, spleen, and thymus (**S1D Fig**). Comparison of the average offspring per nest determined by genotype ratios in litters from DEL-32 *Cdkn1b*^*+/-*^ x *Cdkn1b*^*+/-*^ crosses at early (N1xN1) and later (N6xN6) generations and found allelic ratios that were not significantly different from the expected Mendelian ratios (p = 0.87 and p = 0.78 for early and late, respectively; **S1E Fig**), suggesting that p27 deletion does not result in embryonic lethality.

We performed comprehensive phenotypic assessment to characterize *Cdkn1b*^*+/+*^ and *Cdkn1b*^*-/-*^ females by collecting blood serum, multiple organs, evaluating the weights and histology of two cohorts of female rats, at 4–6 weeks age and 9–16 weeks of age. In the 4–6 week-old cohort, *Cdkn1b*^*-/-*^ rats (n = 5) had significantly larger spleen (p = 0.0038), thymus (p = 0.0082) and brain (p = 0.0091) consistent with the known role of p27 in regulating lymphocyte [18] and neural progenitor cell [28] proliferation. Total body weights, and other organ measurements, including liver, lungs, ovary, and mammary glands were not significantly different compared to *Cdkn1b*^*+/+*^ animals (n = 7) at the 4–6 week timepoint. (**S1F Fig**). In the 9–16 week-old female cohort, the total body weights of *Cdkn1b*^*-/-*^ rats (n = 16) were significantly greater than wild type (n = 20, p = 1.38x10^-5^). Similar at the 9–16 week timepoint, all other organs measured, with the exception of ovaries, were significantly (determined using Welch t-test) larger in *Cdkn1b*^*-/-*^ (KO) rats as compared to *Cdkn1b*^*+/+*^ wild type (WT): mammary gland (n = 17 WT, n = 9 KO, p = 6.5x10^-4^); spleen (n = 20 WT, n = 10 KO, p = 2.1x10^-11^); thymus (n = 20 WT, n = 10 KO, p = 2.2x10^-7^); liver (n = 20 WT, n = 10 KO, p = 2.1x10^-3^); lungs (n = 17 WT, n = 7 KO, p = 1.4x10^-2^), brain (n = 17 WT, = 9 KO, p = 4.8x10^-7^); pituitary gland (n = 19 WT, n = 9 KO, p = 3.2x10^-4^). Homozygotes also had cataracts from birth with 100% penetrance. Histologically, we observed hyperplasia and adenomas in the pituitary glands, greatly enlarged follicles and corpora lutea in ovaries and uteruses in *Cdkn1b*^*-/-*^ rats at 9 weeks of age, however, the thymus, spleen, and lung appeared histologically normal, despite the significantly increased organ size (**S1G Fig**). The overall increase of body weight and several organs in *Cdkn1b*^-/-^ rats is similar to the phenotype reported in *Cdkn1b*^-/-^ mice [18, 23]. Unlike the *Cdkn1b*^-/-^ mice [18, 23], however, the ovaries of *Cdkn1b*^-/-^ rats showed evidence of corpora lutea. As a result, *Cdkn1b*^-/-^ rats have more normal ovarian function than *Cdkn1b*^-/-^ mice. Indeed, we found that 1 out of 6 *Cdkn1b*^-/-^ (F1xF1) females tested was fertile, in contrast to the complete infertility observed in female *Cdkn1b*^*-*/-^ mice [18, 23]. At 12 weeks of age, this *Cdkn1b*^-/-^ female delivered a normal size litter of 14 pups and lactated sufficiently to nourish her pups, indicating that *Cdkn1b* deletion in rats does not ablate the ability to complete pregnancy and lactate. For comparison, *Cdkn1b*^*+/-*^ and *Cdkn1b*^*+/+*^ (F1xF1) females tested for fertility were 100% fertile, as 4 out of 4 females produced a litter at 12 weeks of age, suggesting that fertility in *Cdkn1b*^*-/-*^ is strongly reduced, but not completely absent.

### Characterization of mammary glands of *Cdkn1b*^-/-^ rats

To analyze the effects of p27 loss on mammary epithelial cell morphogenesis and differentiation, we characterized the mammary glands of 9-week-old adult virgin females. Whole mount analyses demonstrated that the homozygous knockout mammary glands at this age already showed a large expansion of alveolar buds (**Fig 1C**), which are the rodent equivalent of the terminal duct lobule unit (TDLU) in the human breast [29]. Analysis of hematoxylin and eosin (H&E) stained mammary gland tissue sections verified that the *Cdkn1b*^-/-^ mammary glands have more terminal end bud structures, covering a larger area of the mammary fat pad compared to wild type (**Fig 1D**). In addition, at this age, the *Cdkn1b*^-/-^ mammary glands show luminal ectasia (dilated milk ducts), a phenotype previously observed in BN rats resistant to E2-induced mammary tumors [30]. The highly developed mammary gland phenotype observed in 9-week old *Cdkn1b*^-/-^ rats is in sharp contrast to the hypoplastic mammary glands observed in intact *Cdkn1b*^-/-^ mice, as well as transplanted *Cdkn1b*^-/-^ mammary epithelium [21]. Contrary to the pituitary gland adenomas, we did not observe any neoplastic changes in the mammary epithelium of *Cdkn1b*^-/-^ rats nor any signs of nuclear abnormalities described in fibroblast cultures derived from *Cdkn1b*^-/-^ mice [31].

To analyze mammary gland morphology and differentiation in further detail, we performed immunofluorescence analysis of smooth muscle actin (SMA) myoepithelial cell and Ki67 proliferation marker, as well as milk casein on tissue sections from *Cdkn1b*^+/+^ and *Cdkn1b*^-/-^ rats at 9 weeks of age. The basal epithelial layer, containing SMA^+^ myoepithelial cells, was intact in *Cdkn1b*^-/-^ rats, but they had significantly greater total numbers of epithelial cells (**Fig 1E**) Despite having significantly more cells in the mammary epithelium, the relative fraction of Ki67^+^ cells did not significantly differ across *Cdkn1b*^+/+^ and *Cdkn1b*^-/-^ genotypes (**Fig 1F**). The morphology of *Cdkn1b*^-/-^ mammary glands resembled pregnancy/lactation-related changes, a phenotype further supported by high expression of milk protein (**Fig 1E** and **1F**) and comparison to mammary glands of ACI rats at multiple stages of gestation (**S2A Fig**).

To assess if the genetic ablation of p27 alters the relative frequencies of mammary epithelial progenitors, we performed Fluorescence Activated Cell Sorting (FACS) analysis using cell type-specific cell surface markers on epithelial populations from rats 9 weeks of age. We used Cd31 and Cd45 to visualize endothelial and hematopoietic cells, respectively, while antibodies against Cd24, Cd29, and Cd49b, as well as Peanut Agglutinin (PNA) were used to quantify the relative frequencies of luminal (Cd24^+^Cd29^low^), basal/myoepithelial (Cd24^+^Cd29^high^; also containing mammary stem cells), and luminal progenitor (Cd24^+^Cd29^low^Cd49b^+^ or Cd24^+^Cd29^low^PNA^+^) sub-populations (**S2B Fig**). The relative frequency of Cd24^+^Cd29^high^ basal/myoepithelial cells did not differ significantly between *Cdkn1b*^*+/+*^ and *Cdkn1b*^*-/-*^ rats, however, the proportion of Cd24^+^Cd29^low^ mature luminal cells was significantly higher in *Cdkn1b*^*-/-*^ rats (**Fig 1G**). By analyzing the expression of Cd49b, a known marker for luminal progenitors in the mouse mammary gland [32], we found that Cd49b was expressed mainly in the Cd29^low^ luminal compartment and the fraction of Cd24^+^Cd29^low^Cd49b^+^ luminal cells was significantly reduced in *Cdkn1b*^*-/-*^ rats (**Fig 1G**). The relative fraction of Cd24^+^Cd29^low^PNA^+^ luminal cells did not statistically differ between the two groups, likely due to the fact that PNA stains a wider range of luminal cells, including alveolar progenitors [33]. We also characterized the mammary glands of 4-6-week-old prepubertal rats to determine if there are any visible differences prior to puberty. Whole mount, H&E, and SMA staining of the mammary glands of 4-6-weeks-old *Cdkn1b*^*+/+*^ and *Cdkn1b*^*-*/-^ animals did not reveal any obvious differences (**S2C Fig**). Similarly, FACS analysis did not detect any significant differences in the relative frequencies of basal, luminal, and progenitor cell populations in this age group (**S2D, S2E,** and **S2F Fig**). Taken together, these data suggest that deletion of p27 leads to the expansion of mature luminal cells with a concomitant decrease in the frequency of luminal progenitors quantified from total mammary epithelial cell populations in post-pubertal rats, resulting in an overall more differentiated mammary epithelium. Because these changes are only observed after puberty, they could be due to differences in hormone levels or in the properties of hormone-responsive mammary epithelial cells.

### Mammary cell transplantation experiments

**Fig 2:**
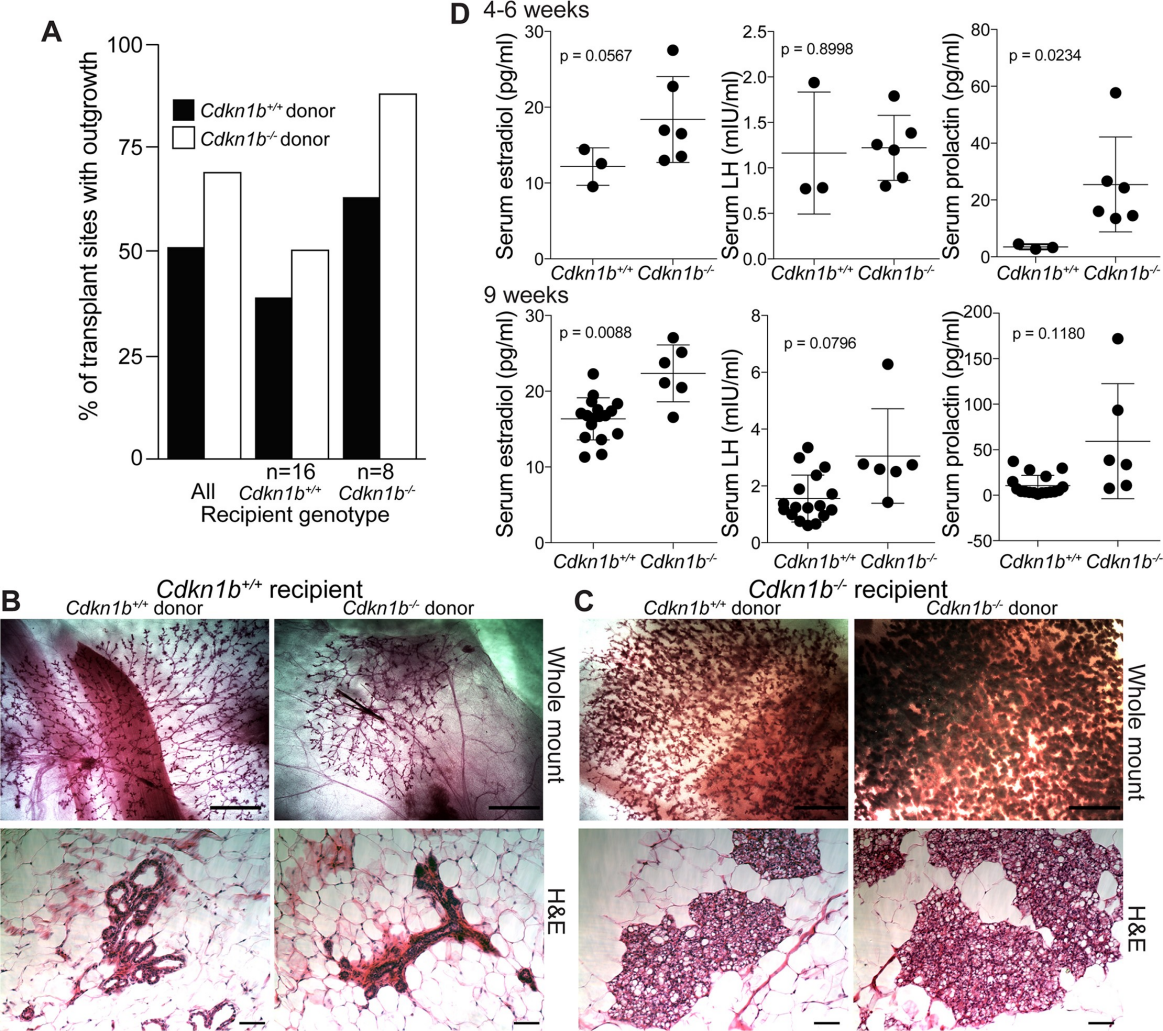
Mammary gland transplants. (A) Percentage of sites with outgrowth from *Cdkn1b*^*+/+*^ and *Cdkn1b*^*-/-*^ donors to *Cdkn1b*^*+/+*^ and *Cdkn1b*^*-/-*^ recipients. (B) Representative whole mounts and H&E staining of transplanted mammary outgrowth from *Cdkn1b*^*+/+*^ and *Cdkn1b*^*-/-*^ donors to *Cdkn1b*^*+/+*^ recipients. Scale bars are 5 mm and 100 μM (H&E), respectively. (C) Representative whole mounts and H&E staining of transplanted mammary outgrowth from *Cdkn1b*^*+/+*^ and *Cdkn1b*^*-/-*^ donors to *Cdkn1b*^*-/-*^ recipients. Scale bars are 5 mm and 100 μM (H&E), respectively. (D) Serum levels of the indicated hormones in Cdkn1b^+/+^ and Cdkn1b^-/-^ females at 4 (top) and 9 (bottom) weeks of age. Error bars represent mean ±SD. P-values determined by t-test.

To determine if the mammary gland development phenotype is intrinsic to the p27-deficient epithelium or controlled by the host, we conducted mammary gland transplantation assays using rats at 4–5 weeks of age (a developmental timepoint prior to the onset of puberty), allowing 6 weeks for epithelial outgrowth. All of the donor and recipient animals were generated by intercrossing heterozygotes from backcross generation N6. Both DEL-32 and DEL-65 lines were used as recipients and donors. There was no difference in graft efficiency between genotype-matched (DEL-32 into DEL-32; or DEL-65 into DEL-65) and genotype-unmatched (DEL-32 into DEL-65 and vice versa) host-recipient combinations (p = 1.0), indicating that both alleles could be used interchangeably at the N6 backcross generation. Equal numbers of mammary epithelial cells from *Cdkn1b*^*+/+*^ and *Cdkn1b*^*-/-*^ donors were grafted on both sides of the inter-scapular white fat pads of recipient rats (*Cdkn1b*^*+/+*^ or *Cdkn1b*^*-/-*^). A total of 10 donors (n = 5 *Cdkn1b*^*+/+*^, n = 5 *Cdkn1b*^*-/-*^) provided cells for 24 recipients (n = 16 *Cdkn1b*^*+/+*^, n = 8 *Cdkn1b*^*-/-*^). After 6 weeks, the inter-scapular white fat pads containing transplanted cells, along with the endogenous, lower abdominal mammary fat pads, were harvested for whole mount analysis. We quantified the overall outgrowth rate based on the inter-scapular fat pad whole mounts. The outgrowth percentages were 39%, 63%, 50%, 88% for transplant groups (donor:recipient) WT:WT (n = 16), WT:KO (n = 8), KO:WT (n = 16), KO:KO (n = 8, respectively. Analysis by standard logistic regression of the binary outgrowth data indicated no significant donor (p = 0.503) or recipient genotype effect (p = 0.272), and no significant effect of the donor-recipient genotype interaction (p = 0.501) on outgrowth incidence (**Fig 2A**). These results indicate that the fraction of stem/progenitor cells contributing to outgrowth and the mammary fat pad microenvironment are not significantly different between *Cdkn1b*^*+/+*^ and *Cdkn1b*^*-/-*^ rats. This is in accordance with the observation that the mammary glands of *Cdkn1b*^*+/*+^ and *Cdkn1b*^*-*/-^ females in the prepubertal cohort are identical, as we grafted tissue from donor animals 4–5 weeks of age, a timepoint well in advance of puberty-related changes in these rats. Interestingly, the appearance of the grafted tissue was markedly different between *Cdkn1b*^*+/+*^ and *Cdkn1b*^*-*/-^ hosts. We found that the grafts always resembled the phenotype of the host’s endogenous mammary glands, regardless of donor genotype, indicating a strong host effect (**Fig 2B** and **2C**). The host effect was consistent across multiple transplantation experiments. The results indicate that loss of p27 does not significantly affect graft success rate, but it does affect mammary gland development at the onset of puberty. Because the grafted tissues receive the same hormonal regulatory stimuli as the endogenous mammary glands, the endocrine environment in female *Cdkn1b*^*-*/-^ rats is likely to be very different from that of the *Cdkn1b*^*+/+*^ animals.

To analyze hormonal differences between *Cdkn1b*^*+/+*^ and *Cdkn1b*^*-*/-^ rats, we performed serum ELISA for E2, prolactin (PRL), luteinizing hormone (LH), and follicle-stimulating hormone (FSH) in both *Cdkn1b*^*+/+*^ and *Cdkn1b*^*-*/-^ animals at 4–6 and 9 weeks of age. FSH levels were undetectable in all samples. The levels of E2 and PRL were significantly higher in the serum of 9-week-old and 4-6-week-old *Cdkn1b*^*-*/-^ rats as compared to *Cdkn1b*^*+/+*^, while LH levels were not statistically different (**Fig 2D**). These results demonstrate that the endocrine environment is significantly different in *Cdkn1b*^*-*/-^ rats, which likely explains the strong host effect observed in the mammary fat pad transplantation assays. The histological abnormalities we observed in the pituitary glands of *Cdkn1b*^*-*/-^ animals may underlie the observed hormonal changes. Consequently, the altered endocrine environment may also contribute to the partial infertility observed by lack of reproduction in 5 out of 6 females tested.

### p27 loss-induced gene expression changes in mammary epithelial cells of post-pubertal rats

**Fig 3:**
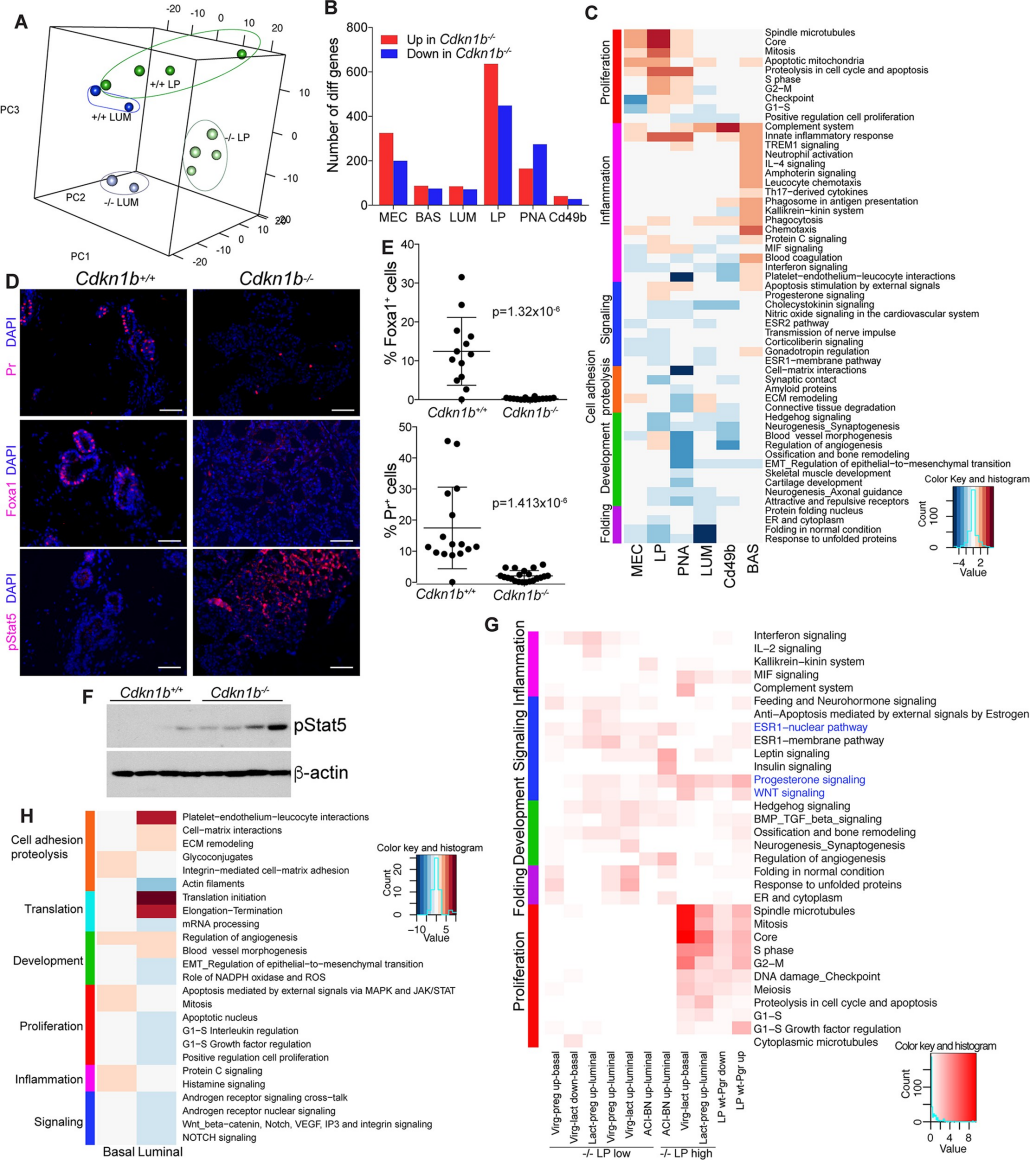
Gene expression changes in *Cdkn1b*^*-/-*^ mammary epithelial cells. (A) PCA plot of luminal (Cd24^+^Cd29^low^) and luminal progenitor (Cd24^+^Cd29^low^Cd49b^+^ and Cd24^+^Cd29^low^PNA^+^) cells. (B) Numbers of significantly up and down regulated genes in *Cdkn1b*^*-/-*^ rats. MEC (all mammary epithelial cells), BAS (basal), LUM (luminal), LP (luminal progenitor). (C) Top enriched process networks (FDR < 0.1) in basal, luminal, and luminal progenitor cells from 9-week-old rats. (D) Immunofluorescence staining analysis of Pr, FoxA1, and pStat5 of *Cdkn1b*^*+/+*^ and *Cdkn1b*^*-/-*^ mammary glands. Scale bar 75 μM. (E) Quantification of the fractions of FoxA1^+^ and Pr^+^ cells. Statistical significance determined using Welch two sample t test of arcsin transformed values. (F) Immunoblot analysis of pStat5 in *Cdkn1b*^*+/+*^ and *Cdkn1b*^*-/-*^ mammary epithelium. (G) Top enriched process networks (FDR < 0.1) in the indicated overlaps. (H) Top enriched process networks (FDR < 0.1) in basal and luminal cells 6-week-old rats.

To characterize molecular mechanisms by which p27-loss leads to altered mammary epithelial cell proliferation and differentiation, we sorted Cd24^+^Cd29^high^ basal/myoepithelial, Cd24^+^Cd29^low^Cd49b^-^PNA^-^ mature luminal cells, and Cd24^+^Cd29^low^PNA^+^ and Cd24^+^Cd29^low^Cd49b^+^ luminal progenitors from 9-week-old virgin *Cdkn1b*^*+/*+^ and *Cdkn1b*^*-*/-^ females and analyzed their gene expression profiles. Luminal and basal cells had the most divergent gene expression patterns due to lineage-specific differences, while differences between *Cdkn1b*^*+/*+^ and *Cdkn1b*^*-*/-^ rats were more pronounced in luminal than in basal cell populations (**S3A Fig**). Principal Component Analysis (PCA) of luminal cell types showed three major clusters, a larger *Cdkn1b*^*+/*+^ mature and luminal progenitor (LP) group, while mature luminal and luminal progenitors from *Cdkn1b*^*-*/-^ rats clustered by themselves (**Fig 3A**). The numbers of significantly differentially expressed genes was the highest in the combined Cd24^+^Cd29^low^PNA^+^ and Cd24^+^Cd29^low^Cd49b^+^ luminal progenitors (**Fig 3B** and **S1 Table**). These data suggest that p27 deletion has the most pronounced effects on luminal progenitors and that these changes are driven by the altered hormonal environment of these rats.

We performed Metacore analysis [34] of the differentially expressed gene lists to identify pathways and networks that are affected by p27 deletion. In basal cells the top up-regulated pathways were chemotaxis, inflammation and other immune-related processes with many chemokines (e.g., Ccl2, Ccl7, Ccl13, Ccl19, Ccr7) overexpressed in *Cdkn1b*^*-*/-^ animals, while top down-regulated pathways included regulation of epithelial-to-mesenchymal transition (EMT) and Notch signaling driven by decreased tuberin, Smad2, Dock1, and Mpdz levels (**Fig 3C)**. Among luminal cells the most significant and highest number of differentially enriched pathways were in the luminal progenitor (LP) fraction, which is consistent with our FACS and gene expression data. Top up-regulated networks in LP are almost all cell proliferation and cell cycle related (e.g., Cdk1, cyclin B, cyclin A, Bub1, Nek2A) implying that p27-deficient luminal progenitors are more proliferative, while down-regulated networks included protein folding (Hsp90, Hsp70, Hsp27), hedgehog signaling (Gli3, Id2, Fgfr2), and hormone-related pathways (Esr1, Creb1) (**Fig 3C)**. Top enriched pathways in mature luminal cells were also mostly cell cycle-related consistent with the expansion of this cell population (**Fig 3C**).

Interestingly, lactation and milk protein-related genes *Wap*, *Csn2*, and *Csn3* exhibited much higher expression in luminal cells of *Cdkn1b*^*-*/-^ rats, while the expression of genes associated with luminal epithelial cell differentiation (e.g., *Esr1*, *Pgr*, and *Foxa1*) were reduced in *Cdkn1b*^*-*/-^ cells (**S1 Table**). In line with this, multicolor-immunofluorescence analysis of mammary gland tissue sections demonstrated significantly fewer Pr^+^ and almost complete absence of Foxa1^+^ mammary epithelial cells in *Cdkn1b*^*-*/-^ rats confirming our RNA-seq data (**Fig 3D** and **3E**). In contrast, the fraction of pStat5^+^ cells was significantly increased in *Cdkn1b*^*-*/-^ mammary glands (**Fig 3D** and **3F**), which is consistent with the pregnancy-related morphogenesis and differentiation in the *Cdkn1b*^*-*/-^ mammary gland, since pStat5 is known to be induced by prolactin [35]. Analysis of these same proteins in mammary glands of pregnant (D11.5), lactating (D9), and involuting (D4) ACI rats showed similar decline in Pr and Foxa1 and increase in pStat5, however, in contrast to the *Cdkn1b*^*-*/-^ glands, proliferation (Ki67^+^ cells) was also decreased in these conditions (**S2A Fig**). Thus, deletion of p27 in ACI rats leads to pregnancy and lactation-associated changes, but with increased proliferation, implying perturbed differentiation and hormonal regulation of luminal progenitors. Interestingly, estrogen-treated BN rat mammary glands also displayed some of these pregnancy/lactation-related changes (**S2A Fig**), which could contribute to their relative resistance to estrogen-induced mammary tumors.

To further delineate the similarity of *Cdkn1b*^*-*/-^ mammary epithelial cells to that of pregnant or hormonally-stimulated animals, we analyzed overlaps between genes high or low in luminal progenitors (LP) of *Cdkn1b*^*-*/-^ rats and different between virgin and G18 pregnant and lactating (day 2) mouse mammary glands [36] as well as mammary glands of ovariectomized mice before and after acute (4–72 hr) progesterone treatment [37]. We also performed RNA-seq of basal and luminal cells from control and E2-treated ACI and BN rats. All overlaps with genes high in luminal progenitors of *Cdkn1b*^*-*/-^ rats were the most significantly enriched in networks related to cellular proliferation including regulation of G1-S and G2-M transition, mitosis and S phase (**Fig 3G**). In contrast, overlaps with genes low in luminal progenitors of *Cdkn1b*^*-*/-^ rats were enriched in networks related to development, hormonal (estrogen and progesterone) and growth factor (Wnt, hedgehog) signaling, and inflammation. These results further imply that luminal progenitors of virgin adult *Cdkn1b*^*-*/-^ rats show pregnancy/lactation-related proliferation and differentiation changes due to their perturbed endocrine environment, and that some of these changes may be due to direct transcriptional regulation by Pr. Interestingly, differences between ACI and BN rats overlapping with differential genes in *Cdkn1b*^*-*/-^ LP cells were enriched in hormonal (estrogen and progesterone) and growth factor (Wnt and Hh) signaling pathways, but not in proliferation-related categories.

### p27 loss-induced gene expression changes in mammary epithelial cells of prepubertal rats

Based on our analysis of *Cdkn1b*^*-*/-^ rats at different ages hormonal and organ-size related changes start to occur at puberty at 6 weeks of age. Thus, to analyze whether p27 deletion causes any molecular differences in mammary epithelial cells in prepubertal animals, we also analyzed the gene expression profiles of Cd24^+^Cd29^high^ basal/myoepithelial (BAS) and Cd24^+^Cd29^high^ luminal epithelial (LUM) cells. We separated this cohort into two age groups, 3–4 weeks (n = 3), when the mammary ducts just start to extend into the fat pad, and 5–6 weeks (n = 2) old animals when the mammary fat pad extension is completed. In the 3–4 week-old group, *Cdkn1b*^*-*/-^ mammary cells did not exhibit clear differences at gene expression level from *Cdkn1b*^*+/*+^ cells, and we did not identify any differentially expressed genes with significant p-value. In the 5–6 week-old cohort we identified 1,182 and 2,717 genes that were significantly differentially expressed in basal and luminal cells, respectively (**S2 Table**). Top up-regulated networks in basal cells are apoptosis (e.g., Mcl1, STAT1, Bcl-3), cell adhesion (e.g., tubulin, actin, Has, elastin), and cell cycle (e.g., Aurora B, Cdc20) related, while top down-regulated pathways are associated with protein folding (e.g., Hsp70, Hsp60) and translation initiation (e.g., 4e-Bbp1, eif3s7) (**Fig 3H**). Top up-regulated pathways in luminal cells include translational regulation with many genes encoding ribosomal proteins (e.g., Rps28, Rpl23a, Rpl28, Rack1, Rps3, Rpl19) and proteins involved in cell adhesion and cell-matrix interactions (e.g., Col1a1, Itga1, biglycan, lumican, Timp3, decorin, Itga4, fibronectin). Many of these upregulated genes are known targets (e.g., fibronectin) or modulators (e.g., decorin, biglycan) of the TGFβ signaling pathway and are also upregulated during EMT [38] implying perturbed luminal epithelial cell differentiation. In line with this, top pathways enriched in genes downregulated in *Cdkn1b*^-/-^ luminal cells include Notch signaling (e.g., Notch1, Hes7, Smad2, Gata-3, Numb) and regulation of cell proliferation (e.g., c-Myc, Gsk3 beta, E2f4, Cdk6). These data suggest that p27 loss in mammary epithelial cells leads to perturbed luminal epithelial differentiation and altered responsiveness to growth factor signaling pathways, and some of these changes occur early in pre-pubertal rats and prior to significant changes in the systemic hormonal environment.

### Direct targets of progesterone receptor (Pr) in *Cdkn1b*^+/+^ and *Cdkn1b*^-/-^ mammary epithelial cells

**Fig 4:**
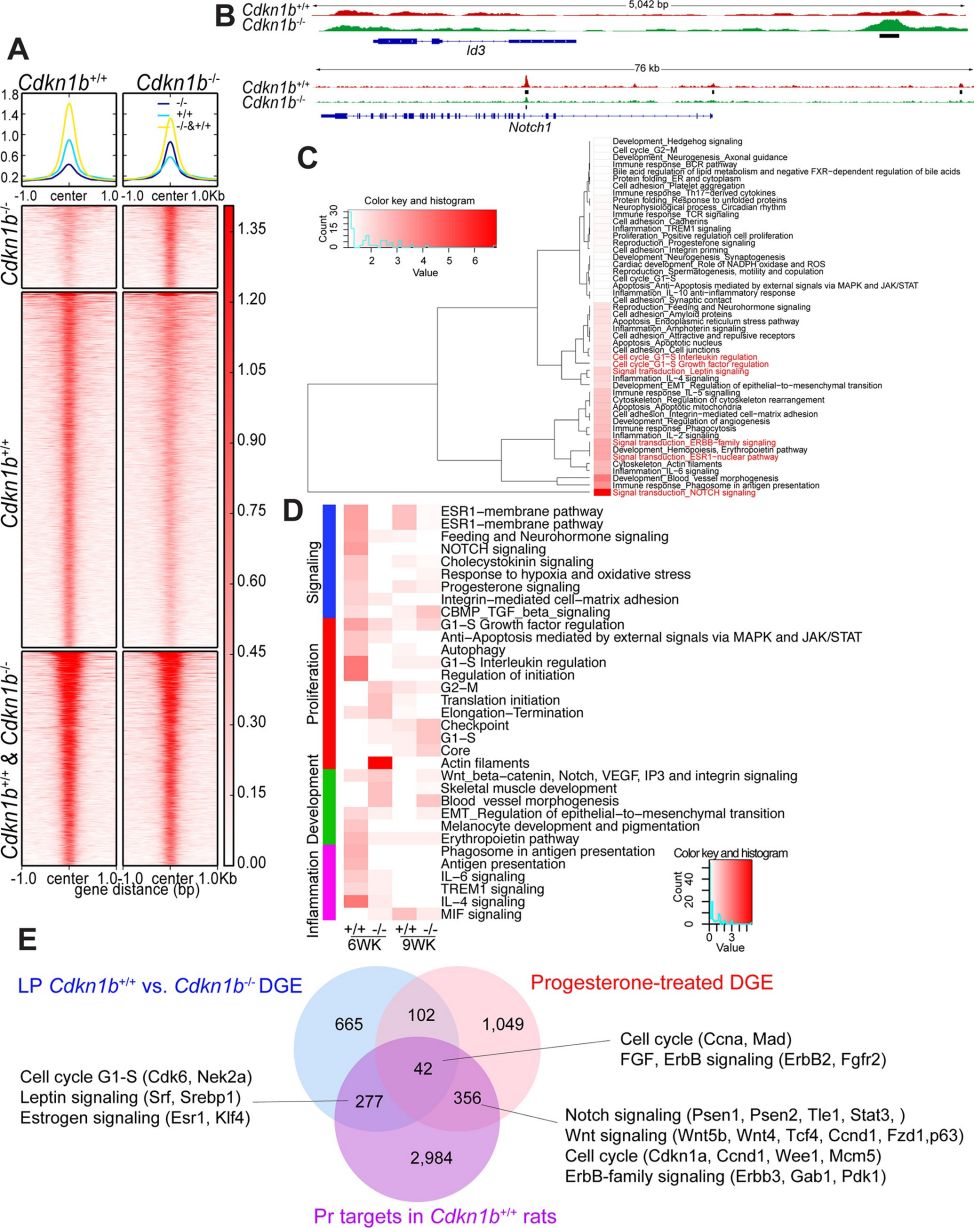
Genomic targets of Pr in mammary epithelial cells of 9-week-old *Cdkn1b*^*+/+*^ and *Cdkn1b*^*-/-*^ female rats. (A) Heatmap depicting Pr peaks common between or unique to *Cdkn1b*^*+/+*^ and *Cdkn1b*^*-/-*^ mammary glands. (B) Gene tracks depicting Pr signal at selected genomic loci in *Cdkn1b*^*+/+*^ and *Cdkn1b*^*-/-*^ mammary epithelial cells. Black bars indicate peak location. (C) Process networks enriched in genes associated with Pr peaks common between *Cdkn1b*^*+/+*^ and *Cdkn1b*^*-/-*^ rats. (D) Process networks enriched in genes differentially expressed in 6 or 9-week-old rats and associated with Pr peak in *Cdkn1b*^*+/+*^ and *Cdkn1b*^*-/-*^ rats. (E) Venn diagram depicting overlap between genes differentially expressed (DGE) in luminal progenitors (LP) of *Cdkn1b*^*+/+*^ and *Cdkn1b*^*-/-*^ rats, acute progesterone treatment in ovariectomized mice, and direct Pr targets in *Cdkn1b*^*+/+*^ rats.

The progesterone receptor is a key transcriptional regulator of mammary epithelial cell differentiation and proliferation [25]. We detected a significant decrease in the fraction of Pr^+^ cells *Cdkn1b*^-/-^ rats (**Fig 3D**) and genes induced by acute progesterone treatment in ovariectomized mice overlapped with genes with altered expression in luminal progenitors of *Cdkn1b*^-/-^ rats (**Fig 3G**), implying that changes in progesterone signaling may be responsible for some of the phenotypic changes we see in *Cdkn1b*^-/-^ rats. To test this hypothesis, we performed ChIP-seq for Pr in mammary epithelial cells from 9 weeks old *Cdkn1b*^+/+^ and *Cdkn1b*^-/-^ females. QC analysis of the number of mapped reads (**S4A Fig**) and total peaks above background (**S4B Fig**) confirmed the quality of the ChIP-seq data. D*e novo* motif search also revealed PR consensus binding sequence as top hit (**S4C Fig**). We identified significant differences in Pr genomic binding between *Cdkn1b*^+/+^ and *Cdkn1b*^-/-^ cells with 3,270 and 760 peaks unique to *Cdkn1b*^+/+^ and *Cdkn1b*^-/-^ cells, respectively, while 1,972 peaks were common (**Fig 4A, S4D Fig**, and **S3 Table**). *Id3* and *Notch1* are examples for differential peaks present in *Cdkn1b*^-/-^ and *Cdkn1b*^+/+^ cells, respectively (**Fig 4B**). Most of the Pr peaks were localized to intergenic regions and introns, with smaller fraction in promoters and this relative peak distribution pattern was essentially the same in *Cdkn1b*^+/+^ and *Cdkn1b*^-/-^ cells (**S4E Fig**). Functional analysis of Pr targets associated with peaks present in both *Cdkn1b*^+/+^ and *Cdkn1b*^-/-^ cells using Metacore revealed that top enriched pathways include Notch, ErbB family, Esr1, and leptin signaling (**Fig 4C**). All these pathways are known to play important roles in mammary epithelial cell differentiation and their direct regulation by Pr confirms its role as a key transcriptional regulator of mammary epithelial cells.

To investigate if differences in Pr chromatin binding could explain some of the differences in luminal gene expression profiles, we integrated our Pr ChIP-seq data with differentially expressed gene lists. Consistent with the known role of progesterone in progenitor cell proliferation and differentiation, many Pr targets unique to *Cdkn1b*^+/+^ cells and differentially expressed between *Cdkn1b*^+/+^ and *Cdkn1b*^-/-^ cells in 9 week-old rats were enriched cell cycle-related genes (e.g., Nek2A, Cyclin A, Cyclin D1, Bub3) and BMP/TGFβ signaling (e.g., Smad6, Smad7, Gli-3) (**Fig 4D** and **S1 Table**). In contrast, Pr targets unique to *Cdkn1b*^-/-^ cells and differentially expressed between *Cdkn1b*^+/+^ and *Cdkn1b*^-/-^ cells in 9 week-old rats were enriched in Erα (e.g., Pr, Srebp1) signaling. Linking Pr targets to genes differentially expressed in 6 week-old animals showed similar results with *Cdkn1b*^+/+^ unique targets enriched in cell cycle (e.g., Bub3, Aurora-B, Cyclin G1, Wee1) and cytoskeleton/muscle differentiation (e.g., Rock2, gelsolin, utrophin, tropomyosin, smooth muscle myosin, filamin C), while *Cdkn1b*^-/-^ unique Pr peaks were enriched in Notch (e.g., GSK3 beta, MAGP2, PI3K reg class IA) and neurohormone (e.g., PPAP2, Lpp3, Ghr) signaling (**Fig 4D** and **S2 Table**).

We also analyzed overlaps between Pr targets and genes induced by acute progesterone treatment or differentially expressed in luminal progenitors of *Cdkn1b*^-/-^ rats to determine to what degree Pr may directly regulate these expression changes. Genes overlapping among all three groups were enriched in networks related to cell cycle and Fgf and ErbB signaling (**Fig 4E**) emphasizing the role of these signaling pathways in the perturbed mammary gland phenotype in *Cdkn1b*^-/-^ rats. Consistent with the known role of Pr, progesterone-induced genes that are also direct Pr targets were enriched in Notch, ErbB family, and Wnt signaling. These results imply that a subset of phenotypic alterations in p27 deficient mammary epithelium is due to alterations in Pr signaling, including altered Pr chromatin binding.

## Discussion

p27 is a cyclin-dependent kinase inhibitor and an important negative regulator of cell proliferation suggesting that it may function as a tumor suppressor [39]. However, the role of p27 in tumorigenesis has been controversial and complex [40, 41]. *Cdkn1b* is not frequently mutated in treatment-naïve human cancers [41] and its homozygous deletion in mice did not lead to malignant growth [18, 23]. However, germline mutations in *Cdkn1b* cause a MEN (multiple endocrine neoplasia) syndrome in humans and rats characterized by pituitary and parathyroid tumors [42]. Furthermore, studies using chemical carcinogens and γ-radiation demonstrated that both heterozygous and complete lack of p27 enhanced tumor development in multiple organs implying that p27 may function as a haplo-insufficient tumor suppressor [43]. Correlating with this, genetic crosses of *Cdkn1b*^*+/-*^ and *Cdkn1b*^*-/-*^ mice with mammary (MMTV-neu) [44] and prostate (Nkx3.1and Pten deficient) [45] tumor models also demonstrated a dosage sensitive effects as *Cdkn1b*^*+/-*^ crosses increased tumorigenesis, while complete deletion of p27 decreased it. Other studies suggested that p27 may have both cell-autonomous and cell-nonautonomous functions and some of this can be independent of its role in cell proliferation [46, 47].

p27 protein levels are prognostic in breast and other cancer types [40]. In breast cancer, lower p27 protein levels are associated with worse overall and disease-free survival in patients with estrogen-receptor positive (ER^+^) tumors [48]. Besides its role in established tumors, p27 may also influence cancer risk potentially via its effects on regulating body size and progenitor cell functions [49]. In line with this we have previously described that p27 may identify hormone-responsive progenitors with proliferative potential in normal human breast tissues, thus, it could potentially be used for risk prediction [3]. Indeed, our prior data in women demonstrated that high Ki67^+^/low p27^+^ and high Ki67^+^/low ER^+^ cell frequencies were significantly associated with a 5-fold higher risk of breast cancer compared to low Ki67^+^/low p27^+^ and low Ki67^+^/low ER^+^ cell frequencies, respectively, among premenopausal women (Ki67/p27: OR = 5.08, 95% CI = 1.43–18.1; Ki67/ER: OR = 4.68, 95% CI = 1.63–13.5) [50]. Thus, *CDKN1B*/p27 may impact both breast cancer risk and disease progression by regulating the proliferation of hormone-responsive progenitors.

In this study we analyzed the role of p27 in mammary gland development by deleting *Cdkn1b* in the ACI rat strain. The rational for generating a *Cdkn1b* knockout in rats was in part due to the closer relatedness of estrogen-dependent mammary tumors in rats to human breast cancers [14] and that germline mutations of *Cdkn1b* cause MEN syndrome in rats [42]. The phenotype of *Cdkn1b*^-/-^ rats showed similarities to that of *Cdkn1b*^-/-^ mice including increased body and organ size with normal morphology and pituitary tumors [18, 23]. However, an important difference is the reduced but not completely absent fertility in female *Cdkn1b*^-/-^ ACI rats compared to female *Cdkn1b*^-/-^ mice that enabled us to analyze mammary gland development in intact rats in contrast to the mammary transplant studies conducted in mice [21, 22]. We found that the mammary glands of prepubertal *Cdkn1b*^-/-^ female rats are essentially indistinguishable from wild type in terms of size, ductal branching, and relative fraction of progenitor and differentiated cells. However, after puberty the mammary epithelium undergoes pregnancy/lactation related changes characterized by extensive proliferation and increase in the total numbers of mammary epithelial cells and milk production. These morphologic changes were accompanied by significant loss of hormone-responsive Pr^+^ and Foxa1^+^ cells. Assessing systemic hormone levels and fat pad transplantation studies demonstrated that the observed alterations are not due to the intrinsic properties of *Cdkn1b*^-/-^ mammary epithelial cells, but caused by the perturbed endocrine environment possibly triggered by pituitary adenomas and hyperplasia. However, our gene expression and Pr chromatin binding pattern profiling by RNA-seq and ChIP-seq, respectively, also suggest changes in the molecular profiles of hormone sensitive cells. Importantly, we detected the most significant differences between *Cdkn1b*^-/-^ and *Cdkn1b*^+/+^ rats in luminal progenitors that demonstrated increased activation of pathways and networks related to cell proliferation, but decreased activity of hormone receptor (e.g., estrogen and progesterone) signaling pathways.

We also analyzed the expression of p27 in E2-induced mammary tumor susceptible ACI and resistant BN strains before and after three weeks of E2 treatment. Interestingly the relative frequency of p27^+^ mammary epithelial cells was significantly higher in untreated ACI compared to BN rats and ACI rats also showed a more obvious increase in proliferation after E2 treatment. These data are in line with our observations in human where we found that women with higher risk of breast cancer (e.g., nulliparous women and *BRCA1/2* mutation carriers) have higher fraction of p27^+^ mammary epithelial cells and also higher fraction of Ki67^+^ proliferative cells [3]. It would be interesting to test in the future whether deletion of p27 affects E2-induced mammary tumor development in ACI and BN rat strains.

In summary, our data demonstrate the power of using genetically engineered rats to study regulators of mammary gland development and breast cancer risk. The ACI rat model particularly could be useful for the functional characterization of genes that influence breast cancer risk due to its susceptibility to estrogen-induced mammary tumors. Our data in *Cdkn1b* knock out ACI rat support a role for p27 as a key regulator of quiescent hormone-responsive luminal progenitors associated with breast cancer risk.

## Materials and methods

### Animal breeding and tissue harvest

All animals were housed and maintained in an AAALAC-approved facility. All animal experiments were approved by the MUSC Institutional Animal Care & Use Committee (IACUC). Rat strains Sprague-Dawley (SD:Hsd; SD), ACI (ACI/Seg), and Brown Norway (BN) females were purchased from Harlan (Envigo). Mutations were generated on the outbred SD genetic background and introgressed onto the ACI inbred genetic background for six generations. As the mutations have been maintained by backcrossing to the ACI inbred genetic background for more than 10 generations, the current, official nomenclature of these congenic rats is ACI.SD-*Cdkn1b*^em1MUSC^ and ACI.SD-*Cdkn1b*^em4MUSC^. To obtain well-controlled cohorts of *Cdkn1b*^+/+^ and *Cdkn1b*^-/-^ rats, we set up heterozygosity crosses at the N1 (early) and N6 (late) backcross generations and selected the desired homozygous (+/+ and -/-) females by genotyping. At 4 or 9 weeks of age, we harvest (lymph node-excised) inguinal/abdominal and all thoracic mammary glands for cellular/molecular analysis. We saved a small section of the inguinal/abdominal mammary gland for histological analysis. These samples were formalin-fixed, paraffin-embedded and processed into histological slides. Another section of the mammary tissue was flash frozen for future molecular analysis. From the remaining fresh tissue (inguinal/abdominal + thoracic) single cell suspensions for FACS analysis were generated as described below. For estrogen treatment of ACI and BN rats silastic^TM^ tubing implants empty or containing 27.5 mg of E2 (Millipore-Sigma), were made and placed surgically into the interscapular region of 9-week-old ACI and BN rats; these implants release hormone continuously for 3 weeks [30].

### Rat germline gene editing using CRISPR-Cas9

The CRISPR-Cas9 technology was implemented following previously established protocols [27]. A sgRNA (5’-GAGTCGAAATTCCACTTGCGC- 3’) was designed to target the protein coding region of Exon 2 of the rat *Cdkn1b* gene. The sgRNA was cloned into the pX330 vector (Addgene) using previously described protocols [51]. In the first microinjection session, this vector was injected at a concentration of 5 ng/μl into the cytoplasm of SD rat zygotes. For a second microinjection session, the sgRNA was prepared by *in vitro* transcription using the Megashortscript T7 kit (Ambion) from template DNA amplified with PCR primers that included the T7 promoter sequence. Purified sgRNA was co-injected with *Cas9* mRNA (Trilink BioTechnologies) into the zygotic cytoplasm at respective concentrations of 200 ng/μl and 500 ng/μl. All microinjections were performed at the MUSC Transgenic and Gene Function Core by following Institutional and IACUC-approved animal research protocols. Genotyping was performed using the following primer combinations to detect *Cdkn1b*^*+/+*^ (WT) and *Cdkn1b*^*-/-*^ (KO) alleles, respectively: WT: 5’-CGGGGAGGAAGATGTCAAA-3’ and 5’-TGGACACTGCTCCGCTAAC-3’, KO: 5’-TGCCGAGATATGGAACAAGC-3’ and 5'-TGGACACTGCTCCGCTAAC-3’.

### Mammary fat pad transplantation

A mammary gland transplantation assay was done essentially as previously published [33]. *Cdkn1b*^*+/-*^ rats of the N6 backcross population (DEL-32 or DEL-65) were intercrossed to obtain *Cdkn1b+/+* and *Cdkn1b-/-* groups. A total of 4 transplantation sessions were done. Donor females were 30–38 days of age, recipients were 30–60 days of age. Mammary organoids were prepared by dissociation of the mammary tissue using Type III Collagenase digestion (Worthington), as previously described [33]. A total of 225K-335K cells, but the same amount for *Cdkn1b*^*+/+*^ (WT) or *Cdkn1b*^*-/-*^ (KO) in a 50% brain homogenate (derived from *Cdkn1b*^*+/+*^ donor) were grafted at either side of the interscapular fat pad. Six to ten weeks after surgery, outgrowth was examined by whole mount analysis of the interscapular white fat pad and compared to a whole mount of the endogenous mammary gland. The whole mount slides were photographed using a digital camera. The outgrowth presence data were analyzed as a binary response by logistic regression. The four transplant groups (WT:WT; WT:KO; KO:WT; KO:KO) form a 2 × 2 factorial design with donor and recipient genotypes as the main effects. Standard logistic regression with two main effects and an interaction term was done to the binary response data.

### Serum hormone measurement

*Cdkn1b*^*+/-*^ rats of the N6 backcross population (DEL-32 or DEL-65) were intercrossed to obtain *Cdkn1b*^*+/+*^ and *Cdkn1b*^*-/-*^ groups. The stage of menstrual cycle was determined by vaginal lavage. Only rats in diestrus or metestrus stage were sacrificed. Blood was collected by cardiac puncture (4-5ml) and centrifuged at 2,500 RPM for 5 minutes, then kept on ice for 90 minutes. After removal of the fibrin clot, serum was transferred two times and centrifuged at 1,200 x g for 5 minutes. The final serum was incubated at 56°C for 30 minutes to deactivate complement and aliquots stored at -80°C. Serum ELISA was performed using undiluted serum and according to manufacturer instructions for Estradiol (Cayman Chemical #582251), Follicle Stimulating Hormone (MyBioSource MBS720215S and Luteinizing Hormone (MyBioSource MBS700807). Serum from wildtype and knockout rats was diluted 1:2 and 1:4, respectively, to obtain readings within the limits of detection for Prolactin measurement (CusaBio CSB-E06881r). Significance was determined using Student’s t-test.

### Mammary epithelial cell purification and FACS

Mammary glands from female *Cdkn1b*^*+/+*^ and *Cdkn1b*^*-/-*^ groups (both N1xN1 and N6xN6) were harvested and dissociated as described [5, 30]. Single cell suspensions were blocked in PBE (PBS with 0.5%BSA, and 2 mM EDTA) and stained at 4°C for 30 minutes with antibodies: CD24-PE (HIS50, BD), CD29-PE/CY7 (HMbeta1-1, AbD Serotec), CD31-APC (TLD-3A12, AbD Serotec), CD45-V450 (OX-1, BD), CD49b-PerCP/Cy5.5 (HMalpha2, Biolegend) and Peanut Lectin(PNA)-FITC (Sigma). Cells were analyzed and sorted using BD FACSAria II SORP UV (Becton Dickinson).

### Whole mount, histology, and multicolor immunofluorescence analyses

Mammary glands from female *Cdkn1b*^*+/+*^ and *Cdkn1b*^*-/-*^ groups (both N1xN1 and N6xN6) were harvested and whole mounts were generated as described previously [30]. The inguinal and abdominal mammary glands were collected, fixed, stained in carmine, dehydrated and cleared in xylene. Samples were imaged on a Nikon Ti/E inverted microscope using Nikon Elements software. Histology and multicolor immunofluorescence analyses were performed as described previously [3]. After heat-induced antigen retrieval in TRIS-EDTA buffer (pH 9), the samples were blocked with 5% goat serum PBS and stained with antibodies against Ki67 (BD550609, 1:50), Milk-specific proteins (Nordic-MUbio, RAM/MSP, 1:200), PR (ab16661, 1:100), FoxA1 (ab23738, 1:200) and phospho-Stat5 (AbCam ab194898, 1:250). Images from the stained sections were obtained by Nikon inverted widefield live-cell imaging system. The percentage of cells for each marker was estimated by counting positive cells and total cells per field from 4 to 6 randomly selected regions using ImageJ 1.45s software. Luminal ectasia was scored by measuring the percentage area of milk protein to mammary epithelium.

### Protein expression analysis

Protein lysates preparation and immunoblotting was performed as described previously [30]. Frozen mammary tissues were homogenized with PowerGen Handheld Homogenizer and lysed in RIPA buffer. Proteins were resolved in SDS-polyacrylamide gels (4–12%) and transferred to PVDF membranes by using a Tris-glycine buffer system. Membranes were incubated with 2.5% milk powder in 0.1% Tween20 in TBS (TBS-T) for 1 h at room temperature and incubated overnight at 4°C with primary antibodies: p27 (1:1,000, BD Biosciences cat#610241) and phospho-Stat5 (AbCam ab194898, 1:1,000) diluted in 2.5% milk TBS-T. The CRISPR-Cas9 generated deletion on the coding exon caused a frame-shift leading to premature stop codons at amino acid 47 (del32bp) and 39 (del65bp). HRP-conjugated goat anti-mouse IgG (Thermo Fisher #62–6520, 1:5,000) was used as secondary antibody. The membranes were developed with Immobilon substrate (EMD Millipore). All blots were also probed with an antibody to β-actin as reference. The p27 antibody used in this study was a mouse monoclonal antibody generated against the full-length mouse protein amino acids 1–197. According to consult with the manufacturer, the exact epitopes are unknown. If the deletions were successful, it is unlikely any protein will be detected, since the deletions are predicted to truncate p27 at amino acid 47 and 39, for DEL-32 and DEL-65 respectively. In addition, aberrant transcripts with premature stop codons are known to be subjected to nonsense-mediated decay, limiting expression of truncated protein products.

### RNA-seq

Total RNA was extracted using the RNeasy Mini Kit (Qiagen). The total RNA was measured by Agilent 2100 Bioanalyzer. RNA-seq libraries were prepared using Clontech Low Input mRNA Library (Clontech SMARTer) v4 kit from less than 10ng of purified total RNA according to the manufacturer’s protocol. The concentrations of finished dsDNA library were measured by Qubit Fluorometer, the size of library fragment was measured by Agilent TapeStation 2200, and RT-qPCR for adapted library molar concentration measurement according to manufacturer’s protocols. Uniquely indexed libraries were pooled in equimolar ratios and sequenced on an Illumina NextSeq500 with Single-End 75bp (SE75) reads by the Dana-Farber Cancer Institute Molecular Biology Core Facilities.

### ChIP-seq

Single cell suspensions were obtained from dissociated mammary organoids, 1 × 10^7^ cells were fixed with fixing buffer (50 mM HEPES-NaOH (pH 7.5), 100 mM NaCl, 1mM EDTA) containing 1% paraformaldehyde (Electron Microscopy Sciences, 15714) and crosslinked for 10 min at 37°C. Crosslinking was quenched by adding glycine to a final concentration of 0.125 M. The cells were washed with ice-cold PBS, harvested in PBS. The nuclear fraction was extracted by first resuspending the pellet in 1 ml of lysis buffer (50 mM HEPES-NaOH (pH 8.0), 140 mM NaCl, 1mM EDTA, 10% glycerol, 0.5% NP-40, and 0.25% Triton X-100) for 10 min at 4°C. Cells were pelleted, and washed in 1 ml of wash buffer (10 mM Tris-HCL (pH 8.0), 200 mM NaCl, 1 mM EDTA) for 10 min at 4°C. Cells were then pelleted and resuspended in 1 ml of shearing buffer (10 mM Tris-HCl (pH 8), 1 mM EDTA, 0.1% SDS) and sonicated in a Covaris sonicator. Lysate was centrifuged for 5 min at 14,000 rpm to purify the debris. Then 100 μl of 10% Triton X-100 and 30 μl of 5M NaCl were added. The sample was then incubated with 20 μl of Dynabeads Protein G (LifeTechnologies,10003D) for 1 h at 4°C. Anti-PR antibody (H-190, Santa Cruz, cat# sc-7208) were added to each tube and immunoprecipitation (IP) was conducted overnight in the cold room. Cross-linked complexes were precipitated with Dynabeads Protein G for 2 hr at 4°C. The beads were then washed in low salt wash buffer (20 mM Tris-HCl pH 8, 150 mM NaCl, 10 mM EDTA, and 1% SDS) for 5 min at 4°C, high salt wash buffer (50 mM Tris-HCl pH 8, 10 mM EDTA, and 1% SDS) for 5 min at 4°C and LiCl wash buffer (50 mM Tris-HCl pH 8, 10 mM EDTA, and 1% SDS) for 5 min at 4°C. DNA was eluted in elution buffer (100 mM sodium bicarbonate and 1% SDS). Cross-links were reversed overnight at 65°C. RNA and protein were digested with 0.2 mg ml−1 RNase A for 30 min at 37°C followed by 0.2 mg ml−1 Proteinase K for 1 h at 55°C. DNA was purified with phenol-chloroform extraction and isopropanol precipitation. ChIP-seq libraries were prepared using the Rubicon ThruPLEX DNA-seq Kit from 1 ng of purified ChIP DNA or input DNA according to the manufacturer’s protocol.

### RNA-seq and ChIP-seq data analyses

Raw RNA-seq datasets were aligned to rat reference rn6 genome using the STAR RNA-Seq aligner (version STAR_2.5.1b) as described previously [52]. The read counts for individual genes were generated using the htseq-count script of the HTSeq framework (version 0.6.1p1) [53] and the rn6 refGene annotation file available at the UCSC Genome Browser. Differentially expressed genes are identified by using DEseq2 [54] with cutoff of p-value < 0.05 and log2fold change > 1, ranked by the statistics. PCA and heatmap visualizations were generated using R software. ChiLin pipeline 2.0.0 [55] was used for QC and raw ChIP-seq data were aligned to rat genome rn6 by Burrows-Wheeler Aligner [56]. MACS2 [57] was used for peak calling and Deeptools [58] was used for generating heatmap plots. Process networks and pathway analyses were performed using Metacore (Thomson Reuters). Gene expression counts from RNA-seq for lactating versus pregnant mouse mammary gland [36] were obtained from series GSE60450. Differentially expressed genes were identified by using DEseq2 [54] with cutoff of p-value < 0.05 and log2fold change > 1. Differentially expressed genes from ovariectomized mice treated with progesterone for 4, 16, 28, and 76 hours were gathered from the supplementary information from Fernandez-Valdivia et al. [37]. For comparison with rat, mouse genes were mapped to rat homolog-associated gene names using the biomaRt R package.

### Statistical analyses

Statistical analyses of *in vitro* and *in vivo* experiments were performed using GraphPad Prism software, using statistical tests indicated in figure legends.

## Supporting information

S1 Fig Deletion of *Cdkn1b* in ACI rats and characterization of p27 knockout rats. (A) UCSC Genome Browser-based plot of exon 2 of the rat *Cdkn1b* gene. The image shows the blat result of the 32bp (DEL-32) and 65bp (DEL-65) deletion mutations in exon 2 (first coding exon), as determined by sequencing of the mutations. (B) Predicted translation of the WT (*Cdkn1b*^*+/+*^) and DEL-32 or DEL-65 (*Cdkn1b*^*-/-*^) rat p27 protein. While the wild type p27 protein has 198 amino acids, the DEL-32 and DEL-65 p27 is predicted to be truncated at amino acid 47 and 39, respectively, and to have a premature stop codon. (C) Genotyping result for 4 (out of 18) progeny of an N1xN1 intercross of heterozygous 32bp deletion carriers. PCR using F1+R1 primers can discriminate between wild type (+/+), heterozygous (+/-), and knockout (-/-). The PCR using primer F2 (located within the deleted interval) verifies absence of exon 2 in the homozygous knockout. (D) Western blot result for protein lysates of the mammary gland, spleen, and thymus from WT and KO (DEL-32), which verifies the lack of full length p27 protein in KO female rats. (E) Average offspring per nest showing genotype ratios in litters from *Cdkn1b+/-* x *Cdkn1b+/-* crosses at early (N1xN1) and later (N6xN6) generations. (F) Measurement of body and organ weights of *Cdkn1b*^*+/+*^ (+/+) and *Cdkn1b*^*-/-*^ (-/-) females at 4–6 weeks (4-6w) and 8+ weeks (8+w, range 8–16) of age. (G) Histology of the indicated organs from 9–10 weeks old *Cdkn1b*^*+/+*^ and *Cdkn1b*^*-/-*^ females (DEL-32). Scale bars are 100 μM.
(TIF)

S2 Fig Mammary glands at different developmental stages. (A) Comparison of mammary glands of *Cdkn1b*^*-/-*^ rats to parental ACI strain at different developmental stages and to BN rats. Hematoxyline-eosine (H&E) staining, immunofluorescence for Sma, Ki67, milk protein, Pr, Foxa1, pStat5 using mammary glands from ACI females at pregnant G11.5, lactation D9, involution D4, and ACI and BN females after 3 weeks of estrogen (E2) treatment, together with *Cdkn1b*^*-/-*^ (DEL-32) rats. Scale bars are 100 μM. (B) Representative FACS analysis profiles of mammary glands from 9-week-old *Cdkn1b*^*+/+*^ and *Cdkn1b*^*-/-*^ females. (C) Whole mounts, H&E, and SMA staining of inguinal/abdominal mammary glands of 4-week-old *Cdkn1b*^*+/+*^ and *Cdkn1b*^*-/-*^ females. Scale bars are 5mm (whole mount) and 75 μM (H&E). (D) Representative FACS analysis of mammary glands from 4-week-old *Cdkn1b*^*+/+*^ and *Cdkn1b*^*-/-*^ females. (E) Frequency of the indicated cell populations in mammary glands of 4-week-old female *Cdkn1b*^*+/+*^ and *Cdkn1b*^*-/-*^ rats. Error bars represent ±SD. Statistical significance determined using Welch two sample t test of arcsin transformed values. (F) Frequency of the indicated cell populations in mammary glands of 6-week-old female *Cdkn1b*^*+/+*^ and *Cdkn1b*^*-/-*^ rats. Error bars represent ±SD. Statistical significance determined using Welch two sample t test of arcsin transformed values.
(TIF)

S3 Fig Gene expression profiles. Spearman correlation between the indicated RNA-seq samples from 9-week-old rats using DESeq2 normalized counts of differentially expressed genes.
(TIF)

S4 Fig Genomic targets of the progesterone receptor. (A) Numbers of total, mapped, and uniquely mapped reads of Pr ChIP-seq data. (B) Numbers of total peaks, peaks 10 and 20-fold above background in Pr ChIP-seq data. (C) Top motifs enriched in Pr ChIP-seq peaks in mammary epithelium of *Cdkn1b*^*+/+*^ rats. (D) Venn diagram depicting numbers of unique and overlapping Pr peaks between *Cdkn1b*^*+/+*^ and *Cdkn1b*^*-/-*^ mammary glands. (E) Genomic location of Pr peaks in *Cdkn1b*^*+/+*^ and *Cdkn1b*^*-/-*^ mammary glands.
(TIF)

S1 Table List of genes differentially expressed in mammary epithelial cells from 9-week-old *Cdkn1b^+/+^* and *Cdkn1b^-/-^* rats. Cell type in which the gene is differentially expressed and whether the gene is a Pr target or not are indicated.
(XLSX)

S2 Table List of genes differentially expressed in mammary epithelial cells from 6-week-old *Cdkn1b^+/+^* and *Cdkn1b^-/-^* rats. Cell type in which the gene is differentially expressed and whether the gene is a Pr target or not are indicated.
(XLSX)

S3 Table List of genes associated with Pr peaks in *Cdkn1b^+/+^* or *Cdkn1b^-/-^* rats. Cell type in which the gene is differentially expressed and whether the gene is a Pr target or not are indicated.
(XLSX)

S4 Table Raw data. List of raw numeric data counts (e.g., weights, immunofluorescence, FACS) corresponding to figures in the manuscript.
(XLSX)
