# Supplementary figures and images for: Deletion of Cdkn1b in ACI rats leads to increased proliferation and pregnancy-associated changes in the mammary gland due to perturbed systemic endocrine environment
Source: PLoS Genet. 2019 Mar 20;15(3):e1008002. doi: 10.1371/journal.pgen.1008002 (PMC6443185; doi:10.1371/journal.pgen.1008002)

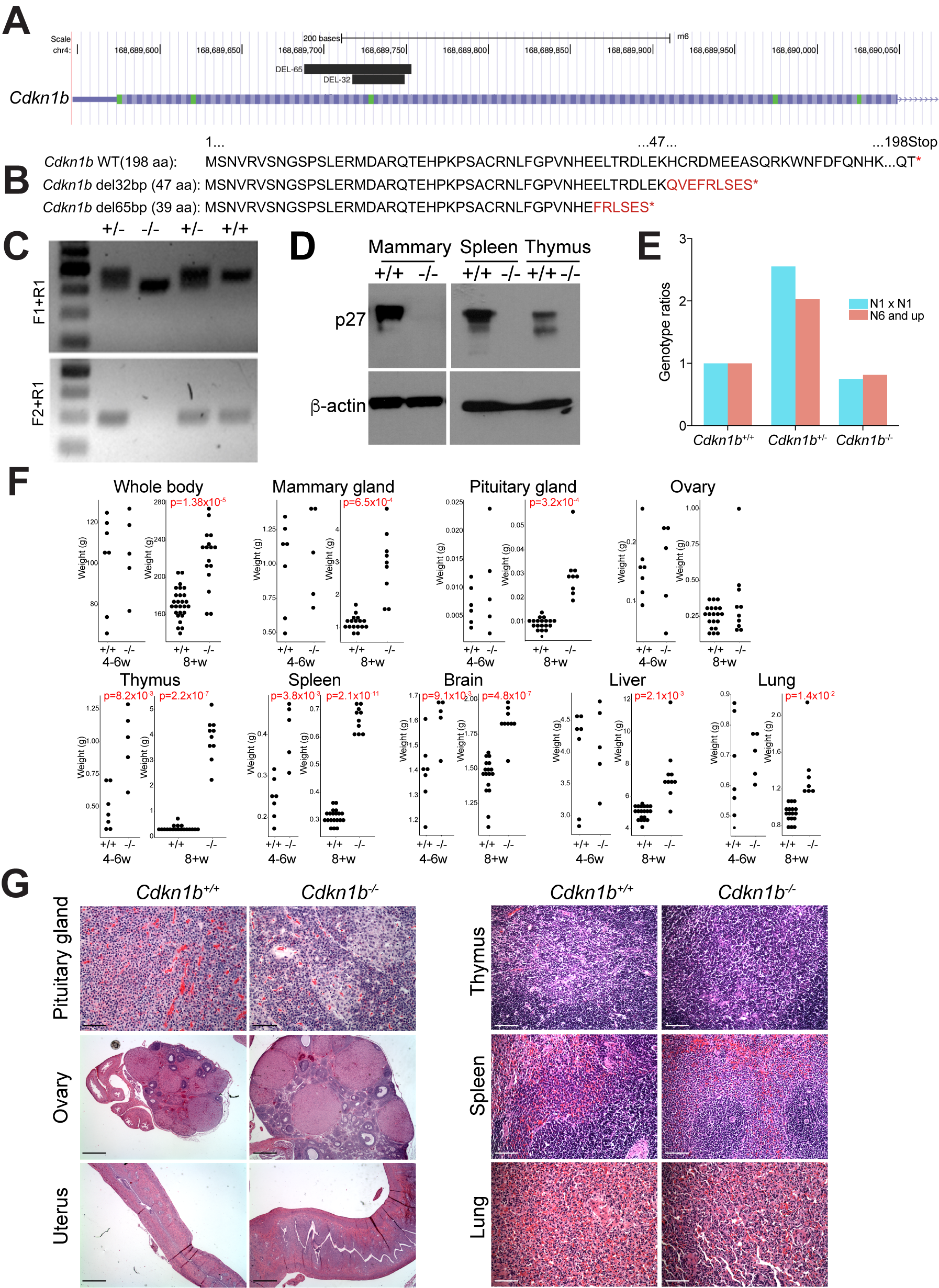

Supplement: S1 Fig — (A) UCSC Genome Browser-based plot of exon 2 of the rat Cdkn1b gene. The image shows the blat result of the 32bp (DEL-32) and 65bp (DEL-65) deletion mutations in exon 2 (first coding exon), as determined by sequencing of the mutations. (B) Predicted translation of the WT (Cdkn1b+/+) and DEL-32 or DEL-65 (Cdkn1b-/-) rat p27 protein. While the wild type p27 protein has 198 amino acids, the DEL-32 and DEL-65 p27 is predicted to be truncated at amino acid 47 and 39, respectively, and to have a premature stop codon. (C) Genotyping result for 4 (out of 18) progeny of an N1xN1 intercross of heterozygous 32bp deletion carriers. PCR using F1+R1 primers can discriminate between wild type (+/+), heterozygous (+/-), and knockout (-/-). The PCR using primer F2 (located within the deleted interval) verifies absence of exon 2 in the homozygous knockout. (D) Western blot result for protein lysates of the mammary gland, spleen, and thymus from WT and KO (DEL-32), which verifies the lack of full length p27 protein in KO female rats. (E) Average offspring per nest showing genotype ratios in litters from Cdkn1b+/- x Cdkn1b+/- crosses at early (N1xN1) and later (N6xN6) generations. (F) Measurement of body and organ weights of Cdkn1b+/+ (+/+) and Cdkn1b-/- (-/-) females at 4–6 weeks (4-6w) and 8+ weeks (8+w, range 8–16) of age. (G) Histology of the indicated organs from 9–10 weeks old Cdkn1b+/+ and Cdkn1b-/- females (DEL-32). Scale bars are 100 μM. (TIF) [file pgen.1008002.s001.tif]

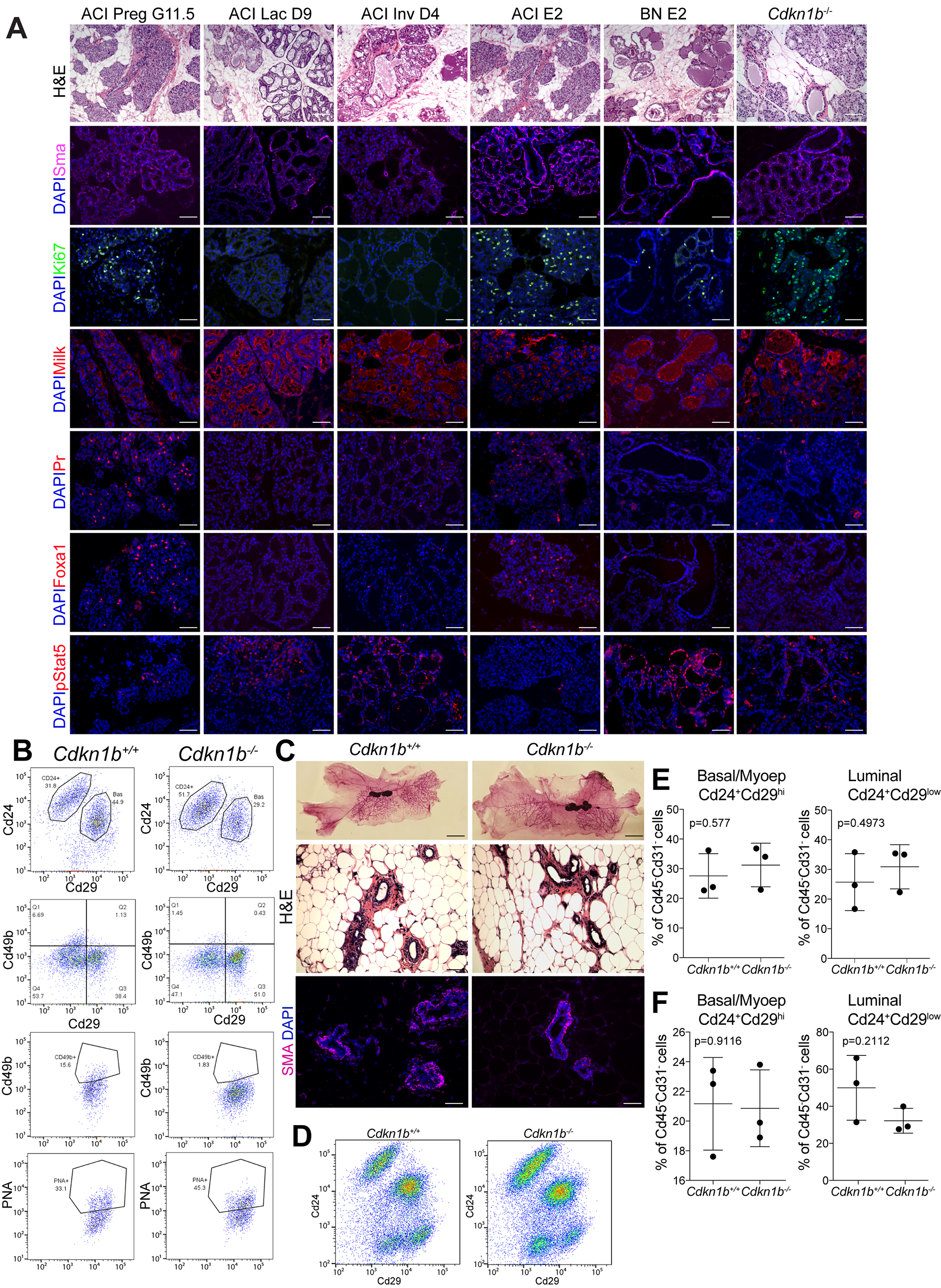

Supplement: S2 Fig — (A) Comparison of mammary glands of Cdkn1b-/- rats to parental ACI strain at different developmental stages and to BN rats. Hematoxyline-eosine (H&E) staining, immunofluorescence for Sma, Ki67, milk protein, Pr, Foxa1, pStat5 using mammary glands from ACI females at pregnant G11.5, lactation D9, involution D4, and ACI and BN females after 3 weeks of estrogen (E2) treatment, together with Cdkn1b-/- (DEL-32) rats. Scale bars are 100 μM. (B) Representative FACS analysis profiles of mammary glands from 9-week-old Cdkn1b+/+ and Cdkn1b-/- females. (C) Whole mounts, H&E, and SMA staining of inguinal/abdominal mammary glands of 4-week-old Cdkn1b+/+ and Cdkn1b-/- females. Scale bars are 5mm (whole mount) and 75 μM (H&E). (D) Representative FACS analysis of mammary glands from 4-week-old Cdkn1b+/+ and Cdkn1b-/- females. (E) Frequency of the indicated cell populations in mammary glands of 4-week-old female Cdkn1b+/+ and Cdkn1b-/- rats. Error bars represent ±SD. Statistical significance determined using Welch two sample t test of arcsin transformed values. (F) Frequency of the indicated cell populations in mammary glands of 6-week-old female Cdkn1b+/+ and Cdkn1b-/- rats. Error bars represent ±SD. Statistical significance determined using Welch two sample t test of arcsin transformed values. (TIF) [file pgen.1008002.s002.tif]

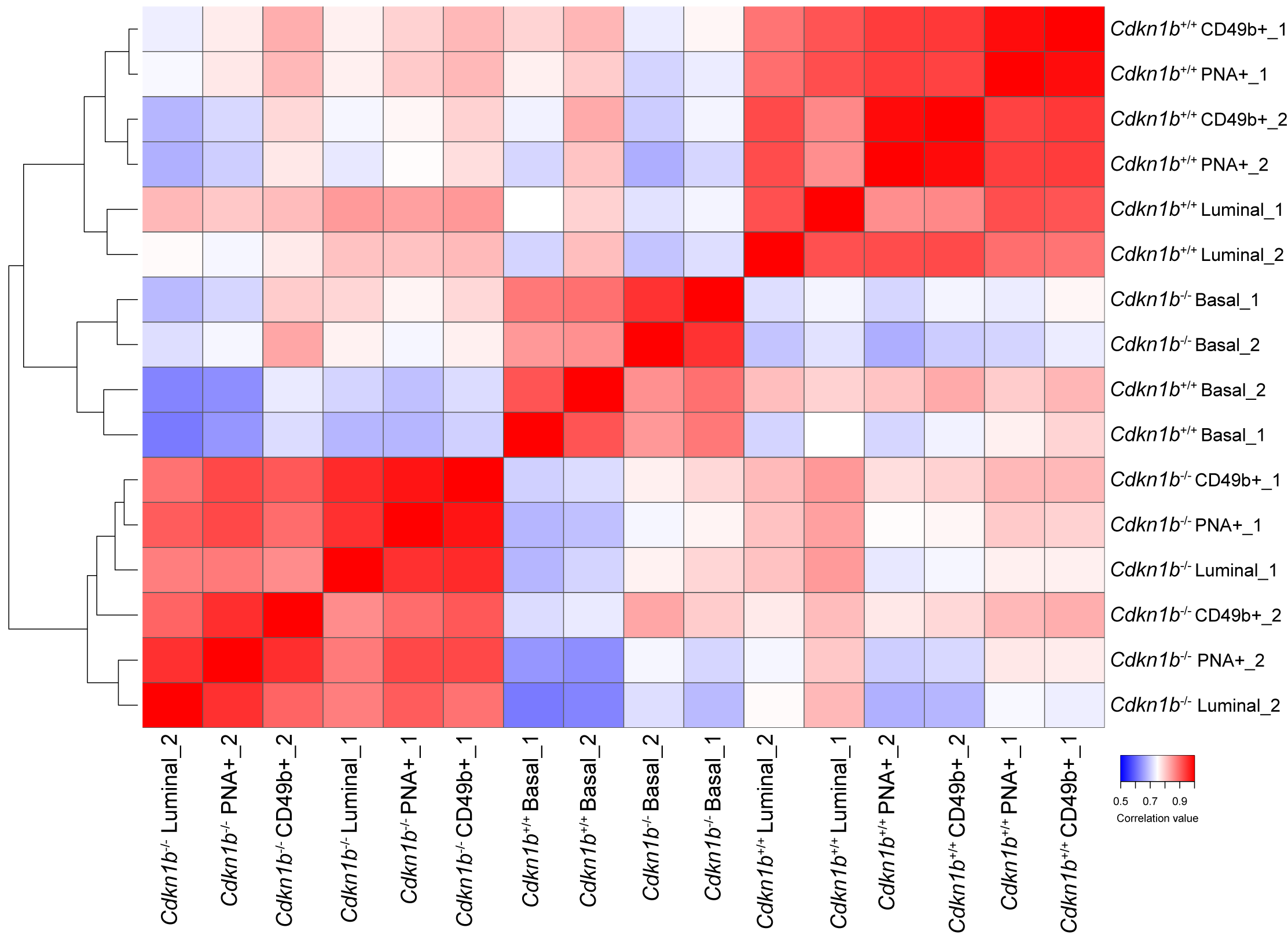

Supplement: S3 Fig — Spearman correlation between the indicated RNA-seq samples from 9-week-old rats using DESeq2 normalized counts of differentially expressed genes. (TIF) [file pgen.1008002.s003.tif]

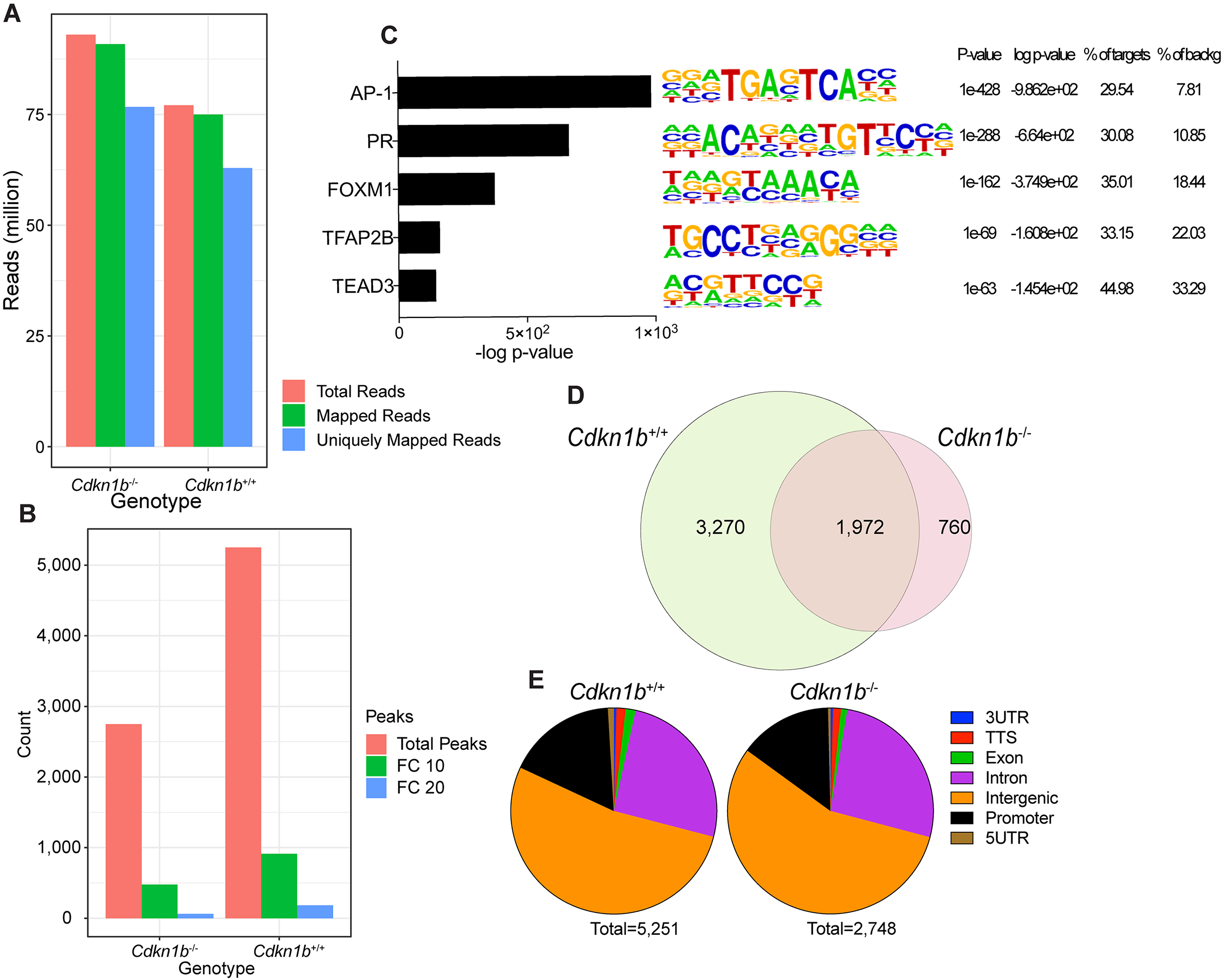

Supplement: S4 Fig — (A) Numbers of total, mapped, and uniquely mapped reads of Pr ChIP-seq data. (B) Numbers of total peaks, peaks 10 and 20-fold above background in Pr ChIP-seq data. (C) Top motifs enriched in Pr ChIP-seq peaks in mammary epithelium of Cdkn1b+/+ rats. (D) Venn diagram depicting numbers of unique and overlapping Pr peaks between Cdkn1b+/+ and Cdkn1b-/- mammary glands. (E) Genomic location of Pr peaks in Cdkn1b+/+ and Cdkn1b-/- mammary glands. (TIF) [file pgen.1008002.s004.tif]
